# Global health crisis and antimicrobial resistance: The need for increased metagenomics studies

**DOI:** 10.1016/j.amsu.2022.104817

**Published:** 2022-11-05

**Authors:** Muritala Abdulkadir

**Affiliations:** Faculty of Pharmaceutical Sciences, University of Ilorin, P.M.B. 1515, Ilorin, 240003, Nigeria; Bayero University Kano, Kano, Nigeria

The impacts of the Coronavirus disease 2019 (COVID-19) still lingers coupled with the current outbreak of monkeypox and other global health crisis, causing a debilitating effect on the global economies, and an increased number of recorded deaths [1]. The annual mortality rate due to antimicrobial resistance (AMR) currently has been estimated to be over 7 million and is hypothesized to reach around 10 million deaths by the year 2050 which can overwhelm the health system together with other global health challenges [2]. The vast usefulness of antibiotics makes antimicrobial resistance a challenge to human health with its impact on global costs and outcomes. Since the discovery of penicillin by Alexander Fleming, antibiotics have been a crucial part of the healthcare system employed in the prevention of child mortality, several invasive surgeries, management of secondary infections, and modern surgeries guided by robots [3].

Naturally, genetic modifications in microbes cause antimicrobial resistance, however, a geometric increase in its prevalence is due to the overexploitation of antibiotics in food, medicine, agriculture, and the environment, making miracle drugs destroy a miracle. The gap in the discovery of novel antibiotics since 1987 calls for other possible mechanisms that can fight against antimicrobial resistance such as the development of rapid diagnostic tests, introduction, and implementation of robust antimicrobial stewardship, removal of growth-promoting antibiotics in food like meat, expansion of surveillance for antibiotic-resistant bacteria, global and interdisciplinary collaboration, and research and development of novel therapeutics [4].

Metagenomics is defined as the characterization of isolated DNA from organisms or free-floating DNA in a certain environment [5]. The technique is an advanced genomics procedure used in the detection of antibiotic resistance in replacement for traditional methods like serial dilution and diffusion, however, the clinical implementation is low but with a prospective anti-infective therapy [6]. Metagenomics studies have been described as the best advancement tools used in the detection of a whole spectrum of AMR from various sources including human gut microbiomes, plants, animals, and the environment [4]. The possibility of one health surveillance in AMR can be improved by metagenomics-based surveillance in the different target populations [7], and an increase in the metagenomics study locally helped the healthcare professionals to make informed and data-driven decisions that ensure better patient outcomes [4].

Diagnostic metagenomics has been identified with the potential for earlier and faster identification of antibiotic-resistant genes in many samples; feces, urine, blood, meat, etc. In addition to specificity and selectivity, diagnostic metagenomics reduced the time to obtain the results from the sample to less than 24 hours [8]. Identifying different microbiome community and their abundance at different environmental and physiological conditions via descriptive metagenomics can be explored in AMR studies and its reduction. Structural metagenomics analysis identifies the mechanism of AMR and the detection of antibiotic reservoir genes to better choose an antibiotic candidate at the clinical level [9]. Functional metagenomics is promising in the discovery of novel antibiotics, antibacterial proteins, and the elucidation of pathways to synthesize antibiotics. The interaction in the ecosystem between the host and microbes as well as among microbes is through functional metagenomics. Metagenomics studies in combination with other “omics” and machine learning tools hold a promising solution to better understanding the AMR at the molecular level to ensure a favorable and personalized antibiotic with a succinct clinical outcome [10]. Globally, surveillance with metagenomics provides data that gives warning on possible e outbreaks of AMR to act in the long-term and guide the development of policies in addition to adequate and effective public health intervention [4].

Provenance and peer review: Not commissioned, externally peer-reviewed.

## Ethical approval

Not applicable.

## Sources of funding

Not applicable.

## Author contribution

Muritala Abdulkadir: Conceptualization, Data collection, writing—original draft, preparation, Writing—Reviewing and Editing.

## Registration of research studies

1. Name of the registry: not applicable
2. Unique Identifying number or registration ID: not applicable
3. Hyperlink to your specific registration (must be publicly accessible and will be checked): not applicable

## Guarantor

Muritala Abdulkadir.

## Consent

Not applicable.

## Declaration of competing interest

The author declares no competing interest.
